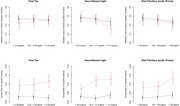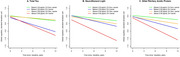# Compounding Effects of APOE4 and Blood Neurodegenerative Biomarkers on Cognitive Decline in Community‐dwelling Older Adults

**DOI:** 10.1002/alz70856_098382

**Published:** 2025-12-24

**Authors:** Ted K.S. Ng, Todd Beck, Patricia A Boyle, Klodian Dhana, Pankaja Desai, Denis A Evans, Kumar B Rajan

**Affiliations:** ^1^ Rush Institute for Healthy Aging, Chicago, IL, USA; ^2^ Rush Alzheimer's Disease Center, Chicago, IL, USA; ^3^ Rush Alz, Chicago, IL, USA

## Abstract

**Background:**

Scarce population‐based data exist on whether APOE4 carrier status accelerates the rate of cognitive decline in non‐demented older adults with elevated neurodegenerative burden.

**Method:**

We analyzed 1,038 community‐dwelling non‐demented older adults (59% Black older adults; 63% women) from the Chicago Health and Aging Project (CHAP), a 20‐year prospective biracial cohort study. We examined the modifying effects of the APOE4 carrier status on the prospective associations of serum neurodegenerative biomarkers with global cognitive decline using a mixed‐effects regression model, adjusting for demographics and chronic health conditions. Statistical analyses were conducted from June 2024 to January 2025. Exposure were APOE4 carrier status and serum biomarker levels for total tau (t‐tau), neurofilament light (NfL) chain, and glial fibrillary acidic protein (GFAP) measured with Quanterix's Neuroplex kit at baseline. Cognitive decline was calculated from composite global cognition scores across study waves.

**Result:**

Of 1,038 participants included, the mean age was 77.1 (SD= 5.9) and had an education of 12.8 (SD= 3.4) years. Higher levels of blood‐based neurodegenerative biomarkers, i.e., t‐tau, NfL, and GFAP, were associated with a faster rate of cognitive decline among APOE4 carriers than non‐carriers. Specifically, compared to non‐carriers, the annual rates of cognitive decline for APOE4 carriers per 1 log_10_ unit higher levels in t‐Tau and GFAP were accelerated by β (SD, *p*‐value)= ‐0.0323 (0.0162,0.0463) and ‐0.0728 (0.0302,0.0162), respectively. Similarly, compared to the non‐carriers and lower NfL tertile, APOE4 carriers with middle and upper tertiles of NfL levels also experienced accelerated cognitive decline, ‐0.0439 (0.0159,0.0058) and ‐0.0321 (0.0179,0.0732), respectively.

**Conclusion:**

In a biracial cohort of community‐dwelling older adults having three different serum neurodegenerative biomarkers and APOE4 carrier status simultaneously examined in the same study, we showed higher neurodegeneration (t‐tau), axonal injuries (NfL), and reactive astrocytes and neuroinflammation (GFAP) accelerated cognitive decline in genetically susceptible APOE4 carriers. Hence, these findings highlight the detrimental role of APOE4 in exacerbating neurodegenerative processes, with not only significant implications for understanding and tracking the progression of neurodegenerative diseases, but also a call for inclusivity of APOE4 status in scientific investigations and in clinical trials.